# Association of a Targeted Population Health Management Intervention with Hospital Admissions and Bed-Days for Medicaid-Enrolled Children

**DOI:** 10.1001/jamanetworkopen.2019.18306

**Published:** 2019-12-27

**Authors:** David M. Rubin, Chén C. Kenyon, Douglas Strane, Elizabeth Brooks, Genevieve P. Kanter, Xianqun Luan, Tyra Bryant-Stephens, Roberto Rodriguez, Emily F. Gregory, Leigh Wilson, Annique Hogan, Noelle Stack, Kathleen Ward, Joan Dougherty, Rachel Biblow, Lisa Biggs, Ron Keren

**Affiliations:** 1 PolicyLab, Children’s Hospital of Philadelphia, Philadelphia, Pennsylvania; 2 Perelman School of Medicine, University of Pennsylvania, Philadelphia; 3 Leonard Davis Institute of Health Economics, University of Pennsylvania, Philadelphia; 4 Department of Pediatrics, Children’s Hospital of Philadelphia, Philadelphia, Pennsylvania; 5 Center for Pediatric Clinical Effectiveness, Children’s Hospital of Philadelphia, Philadelphia, Pennsylvania; 6 Dell Medical School, The University of Texas, Austin; 7 Compass Care Program, Children’s Hospital of Philadelphia, Philadelphia, Pennsylvania; 8 Primary Care, Children’s Hospital of Philadelphia, Philadelphia, Pennsylvania; 9 Press Ganey, Philadelphia, Pennsylvania

## Abstract

**Importance:**

As the proportion of children with Medicaid coverage increases, many pediatric health systems are searching for effective strategies to improve management of this high-risk population and reduce the need for inpatient resources.

**Objective:**

To estimate the association of a targeted population health management intervention for children eligible for Medicaid with changes in monthly hospital admissions and bed-days.

**Design, Setting, and Participants:**

This quality improvement study, using difference-in-differences analysis, deployed integrated team interventions in an academic pediatric health system with 31 in-network primary care practices among children enrolled in Medicaid who received care at the health system’s hospital and primary care practices. Data were collected from January 2014 to June 2017. Data analysis took place from January 2018 to June 2019.

**Exposures:**

Targeted deployment of integrated team interventions, each including electronic medical record registry development and reporting alongside a common longitudinal quality improvement framework to distribute workflow among interdisciplinary clinicians and community health workers.

**Main Outcomes and Measures:**

Trends in monthly inpatient admissions and bed-days (per 1000 beneficiaries) during the preimplementation period (ie, January 1, 2014, to June 30, 2015) compared with the postimplementation period (ie, July 1, 2015, to June 30, 2017).

**Results:**

Of 25 460 children admitted to the hospital’s health system during the study period, 8418 (33.1%) (3869 [46.0%] girls; 3308 [39.3%] aged ≤1 year; 5694 [67.6%] black) were from in-network practices, and 17 042 (67.9%) (7779 [45.7%] girls; 6031 [35.4%] aged ≤1 year; 7167 [41.2%] black) were from out-of-network practices. Compared with out-of-network patients, in-network patients experienced a decrease of 0.39 (95% CI, 0.10-0.68) monthly admissions per 1000 beneficiaries (*P* = .009) and 2.20 (95% CI, 0.90-3.49) monthly bed-days per 1000 beneficiaries (*P* = .001). Accounting for disproportionate growth in the number of children with medical complexity who were in-network to the health system, this group experienced a monthly decrease in admissions of 0.54 (95% CI, 0.13-0.95) per 1000 beneficiaries (*P* = .01) and in bed-days of 3.25 (95% CI, 1.46-5.04) per 1000 beneficiaries (*P* = .001) compared with out-of-network patients. Annualized, these differences could translate to a reduction of 3600 bed-days for a population of 93 000 children eligible for Medicaid.

**Conclusions and Relevance:**

In this quality improvement study, a population health management approach providing targeted integrated care team interventions for children with medical and social complexity being cared for in a primary care network was associated with a reduction in service utilization compared with an out-of-network comparison group. Standardizing the work of care teams with quality improvement methods and integrated information technology tools may provide a scalable strategy for health systems to mitigate risk from a growing population of children who are eligible for Medicaid.

## Key Points

**Question:**

Is a targeted population health management intervention developed for children enrolled in Medicaid and cared for in a large pediatric health system associated with changes in hospital admissions or bed-days?

**Findings:**

In this quality improvement study using difference-in-differences analysis of Medicaid-enrolled children, children exposed to an integrated population health management program experienced a reduction of 0.39 monthly admissions and 2.20 monthly bed-days per 1000 children compared with similar children in the community who were not exposed to the program. Annualized, these differences could translate to a reduction of 3600 bed-days for a population of 93 000 children eligible for Medicaid.

**Meaning:**

Mobilizing interdisciplinary care teams for targeted children with high risk and spreading registry-based information technology tools across a Medicaid population may provide a scalable strategy for other health systems that aim to improve the value of services provided to this population.

## Teaser

This quality improvement study estimates the association of a targeted population health management intervention for children eligible for Medicaid with changes in monthly hospital admissions and bed-days.

## Introduction

A shift in dependent health insurance coverage across the United States in recent years is creating challenges for pediatric health systems to deliver efficient and cost-effective care. Despite an improving economy, Medicaid and Children’s Health Insurance Program growth remains strong, as many low-income and moderate-income families are turning to these programs as a less costly and more comprehensive alternative to the high-deductible and relatively unaffordable coverage options offered by parents’ employers.^1,2^ The overall proportion of children covered by Medicaid remains near 40%.^3^ Medicaid now provides reimbursement for more than half of nonneonatal pediatric hospitalizations.^4^ Such hospitalizations often occur at lower reimbursement rates than commercial insurance, presenting a challenge to financial models for pediatric care.^5^

Pediatric health systems are responding to these new challenges according to local market conditions. For hospitals increasingly serving as the safety net for pediatric tertiary care, capacity challenges for specialty care patients often occur in the context of increasing numbers of children enrolled in Medicaid visiting their emergency departments and requiring inpatient hospitalization. For others, these capacity challenges have been further exacerbated by children with complex medical conditions increasingly relying on the limited numbers of specialty care clinicians who tend to aggregate to children’s hospitals and large pediatric systems.^6,7,8^ Often qualifying for Medicaid coverage on the basis of disability, these children account for an increasing proportion of inpatient days within these hospital systems. Between 2004 and 2009, the number of bed-days at children’s hospitals attributed to children with medical complexity increased by 30%, compared with a 10% increase among other children.^9^ Finally, as the Medicaid program has grown in size, many systems are facing increasing pressure from payers to engage in risk contracting that requires value-based care for Medicaid recipients.^10,11,12,13^

In this context, many pediatric health systems and hospitals are in need of population-directed initiatives to reduce preventable admissions and shorten lengths of stay among children enrolled in Medicaid without compromising quality of care. To date, the evidence of the success of such initiatives has been mixed, particularly for organizations assuming accountable care contracts.^14,15^ In pediatrics, few rigorous evaluations of system-level approaches to population management seeking to decrease use of inpatient services exist.^16^

The purpose of this study was to assess the association of a targeted population health management program with changes in admissions and bed-days among children enrolled in Medicaid and attributed to a primary care network in a large pediatric health system. We used a quasi-experimental design to evaluate the population health program, assessing differences in hospital stays between children who were enrolled in Medicaid and in-network (ie, eligible for the care management programs because of receipt of care within the health system’s primary care network) and those who were out-of-network (ie, received primary care elsewhere in the community).

## Methods

### Institutional Setting and Participants

The site of the population management program was a large mid-Atlantic children’s hospital and primary care network with 31 practices. The health system serves approximately 500 000 children annually, more than half of whom are served in primary care. This study was exempt from institutional review board oversight as analyses were conducted under operational program improvement initiatives.

The target population for the intervention consisted of children enrolled in Medicaid aged 0 to 18 years who were in-network patients. This population was defined between January 1, 2014, and June 30, 2017. In each month, patients were considered in-network if they had visited a physician or nurse practitioner in the health system’s primary care network during the prior 24 months.

The comparison population (ie, out-of-network group) consisted of children in the region who were enrolled in Medicaid but had not seen an in-network primary care clinician in the past 24 months. This population was estimated for each month in the study period using publicly available, monthly Medicaid enrollment files for the 5-county region in the state in which the hospital was located, excluding all in-network patients.

### Intervention

**Figure 1.**
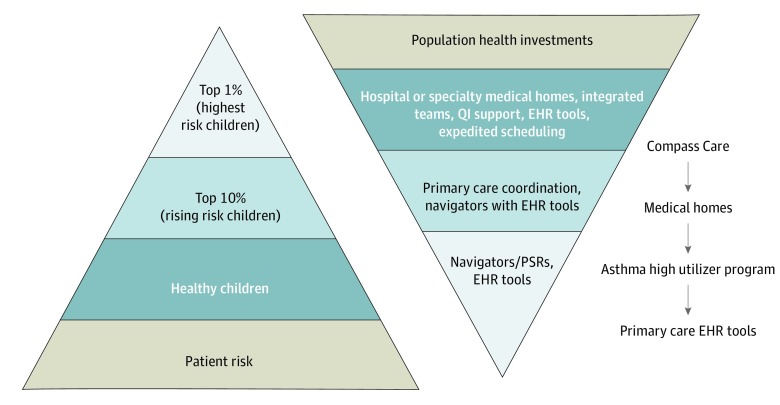
Conceptual Model of Tiered Intervention Strategies for a Medicaid-Focused Population Management Program Given limited resources, interventions were created for each level of rising risk in the population. Alongside the conceptual model are the chosen interventions across different risk groups. The Methods section contains fuller descriptions of each of these programs. EHR indicates electronic health record; PSR, physician for social responsibility; and QI, quality improvement.

The intervention served patients in the health system’s primary care network and targeted population management interventions across a spectrum of risk groups within that population. We used a framework emphasizing tiered distribution of services based on 3 risk profiles, as follows: healthier children, children with rising risk, and children with the highest risk^17^ (Figure 1). Clinicians and staff were provided with registry and reporting tools within the electronic health record (EHR) to respond to patients’ emergency department and hospital utilization, to schedule timely well-child care visits and immunizations, and to proactively prepare for upcoming specialty care visits. These tools stratified patients by risk and alerted clinicians to sentinel emergency department visits in the area, allowing the team to prioritize follow-up scheduling or proactive telephonic outreach. Next, more targeted integrated care teams were developed for children with higher risk, including medically complex children who were either technology dependent or had 2 or more complex chronic conditions (CCCs)^18^ and demonstrated an increased intensity of health care utilization as well as children who were frequently hospitalized for asthma.

Three integrated care team programs were developed to serve high-risk populations. The launch date for the population health management programs and reporting tools was July 1, 2015. The most resource-intense program was a hospital-based care management program for 100 children within the practice network who had multiple complex medical conditions, technology dependence, and recurrent inpatient admissions. Those with the greatest service utilization were provided a team of clinicians, nurses, and social workers to actively manage their care across inpatient and ambulatory settings. The second program, supported in part through the health system’s participation in a Centers for Medicaid & Medicare Innovation Award, created a system of primary medical home management, which included new care coordinators and a standardized care coordination practice, for 735 children enrolled in Medicaid with multiple comorbid complex conditions in 6 of the 31 primary care practices in the network that had high concentrations of children with medical complexity. The third program was a multidisciplinary population health program targeting children with asthma. This program created a bundle of integrated services for children enrolled in Medicaid within the health system’s primary care network who had 3 or more asthma-related hospitalizations in the preceding year and expanded the capacity of a community health worker–staffed home visitor program.^19,20^ This program included personalized bedside education, filling discharge prescriptions on site, expedited outpatient follow-up with an allergist or pulmonologist, and referral to the community health worker home visitor program.

The population management approach was similar for the 3 programs. Each program integrated teams of clinicians, nurses, and other health care professionals across the care continuum who worked longitudinally under a quality improvement framework to develop interventions to reduce admissions and overall hospital bed-days among their populations. To do so, each program was provided a dedicated improvement advisor who, through an Institute for Healthcare Improvement framework, moderated an improvement process in which care teams created driver diagrams that identified changes to staff workflow that would create more proactive care delivery and affect subsequent utilization outcomes.^21^ This process allowed care teams to prioritize proactive clinical workflows that would mitigate risk for admission among their patients. Next, EHR, registry, and reporting tools were developed to distribute workflows among integrated care teams that were responsive to their quality improvement plans. From workflow driver diagrams, the teams also developed longitudinal data tracking resources to monitor process and outcome metrics longitudinally and inform plan-do-study-act cycles for program improvement.

### Outcomes

The primary outcomes were monthly hospital admissions and total days of inpatient occupancy (ie, bed-days). Because the distribution of patient bed-days was highly right-skewed owing to a small number of patients with very long lengths of stay, we computed bed-days based on length of stay winsorized at the 99th percentile (in this sample, 55.7 days) to minimize the effect of extreme outliers.

We calculated total monthly admissions and bed-days for in-network and out-of-network patients. We excluded admissions and bed-days from the neonatal intensive care unit because those children could not have been enrolled in the population management solutions without prior primary care exposure. To estimate in-network and out-of-network rates of service utilization for each month, monthly admission and bed-day counts were converted to rates per 1000 Medicaid beneficiaries in each population.

### Covariates

In base models of trends over time, we included monthly indicator variables to account for seasonal variation in admissions and bed-days during the year. Adjusted models accounted for differences across the admitted populations in age, sex, and race/ethnicity.

To control for potential differential changes in the case mix of the intervention and comparison groups during the study period, adjusted models also accounted for the proportion of in-network and out-of-network Medicaid enrollees diagnosed with at least 2 CCCs, where CCCs are defined as “any medical condition that can be reasonably expected to last at least 12 months (unless death intervenes) and to involve either several different organ systems or 1 organ system severely enough to require specialty pediatric care and probably some period of hospitalization in a tertiary care center.”^18^ The classification system was updated and validated for this analysis using the health system’s EHR of all *International Statistical Classification of Diseases and Related Health Problems, Tenth Revision *(*ICD*-*10*) codes given to patients over the 3-year period. In each month, the comparison population of medically complex children included out-of-network patients with at least 2 CCCs who were enrolled in Medicaid, were aged 0 to 18 years, resided in the catchment area, and had visited an in-network subspecialty practice but had not visited the system’s primary care clinics in the past 24 months. Because health information was not available from Medicaid enrollment files, this comparison group served as a proxy for Medicaid beneficiaries with multiple CCCs who are out-of-network and therefore unexposed to the health system’s population health intervention.

### Statistical Analysis

We computed the distributions of demographic characteristics (ie, age, sex, and race/ethnicity) and the number of CCCs of in-network and out-of-network study participants. Children with medical complexity were considered a time-dependent variable because the population of children with medical complexity attributed to the in-network practices increased at a greater rate than that attributed to the out-of-network practices during the study period (eFigure in the Supplement).

To estimate the association of the population health intervention with admissions and bed-days, we used the quasi-experimental method known as *difference-in-differences*.^22^ This method is an improvement over a simple pre-post design because of its use of a comparison group. First, we graphically compared monthly admission and bed-day rates between the in-network and out-of-network populations using Lowess estimates of trend to evaluate the consistency in trend between the in-network and out-of-network groups during the preintervention period of January 2014 to June 2015.^23^ These estimates were adjusted using monthly dummy variables to account for naturally occurring variations in admissions and bed-days during the year.

To obtain difference-in-differences estimates, we compared changes in utilization among the in-network intervention group with changes in utilization among the out-of-network comparison group. Formally, the statistical model estimated was Y_g,t_ = β_0_ + β_1_ 1[intervention]_g,t_ + β_2_ 1[post]_g,t_ + β_3_ 1[intervention]_g,t_  × 1[post]_g,t_ + Γ' [covariates]_g,t_ + ε_g,t_, where Y is the outcome of interest (admissions or bed-days rate); 1[intervention] indicates whether the group is the in-network intervention group (ie, 1 for the intervention group and 0 otherwise); 1[post] indicates whether the observation date is on or after the start of the intervention (ie, 1 for dates on or after June 1, 2015, and 0 otherwise); [covariates] is a vector of covariates; ε is the disturbance term; g indexes the group (ie, in-network intervention or out-of-network comparison); and t indexes the time in months.

We tested for parallel preintervention trends by estimating the unadjusted model with the addition of the group indicator interacted with a linear time trend. Rejection of the null hypothesis that the population coefficient on the group-time trend interaction term is 0 indicated rejection of the parallel trends assumption.

We obtained difference-in-differences estimates in both unadjusted and adjusted models, reported with 95% CIs for monthly admissions and bed-days per 1000 Medicaid beneficiaries. The unadjusted model included only monthly dummy variables, while the fully adjusted models adjusted for monthly measures of the proportion of patients who were of female sex, black race or Latino ethnicity, or had at least 2 CCCs to account for differential growth in the number of children who were in-network and presented with increasingly complex medical conditions over time.

All analyses were conducted using Stata version 14 (StataCorp). Statistical significance was set at *P* < .05, and all tests were 2-tailed.

## Results

Between January 1, 2014, and June 30, 2017, a mean of 93 365 children enrolled in Medicaid were designated as in-network to the health system’s primary care practices each month. This cohort increased by 32.1% during the study period, from 76 258 children in January 2014 to 100 767 children in June 2017. The number of children with medical complexity who received in-network care increased by 44.1%, from 1131 children in January 2014 to 1632 children in June 2017, surpassing the increase among out-of-network children (eFigure in the Supplement). Of the 99 256 Medicaid-enrolled children seen at in-network primary care practices in January 2017, 13 122 (13.2%) were aged 1 year or younger, 25 184 (25.4%), 2 to 5 years; 31 971 (32.2%), 6 to 11 years; and 28 979 (29.2%), 12 years or older. Overall, 53 455 children (53.9%) were black individuals, 12 223 (12.3%) were Hispanic individuals, and 2316 (2.3%) had multiple CCCs. The out-of-network population of children enrolled in Medicaid increased by 13.8% during the same period, from 198 548 in January 2014 to 225 989 in June 2017.

During the study period, a total of 25 460 children in our sample were admitted to the health system’s hospital, accounting for 43 555 admissions (Table 1). Approximately one-third of these patients (8418 [33.1%]) were from in-network practices; the remainder were out-of-network (17 042 [67.9%]). Admitted in-network patients were similar in sex compared with admitted out-of-network patients (3869 [46.0%] vs 7779 [45.7%] girls) but differed significantly in age (eg, aged ≤1 year, 3308 [39.3%] vs 6031 [35.4%]; aged 2-5 years, 2120 [25.2%] vs 3690 [21.7%]). A greater proportion of in-network admitted patients were black individuals (5694 [67.6%] vs 7167 [41.2%]). Admitted out-of-network patients had higher numbers of CCCs than in-network patients (≥2 CCCs, 2395 [14.1%] vs 910 [10.8%]), although they were similar as a proportion of all admissions (7929 [29.0%] vs 4500 [27.8%]).

**Table 1.** Characteristics of Admitted Medicaid Patients by Receipt of In-Network Primary Care, January 2014 to June 2017

Characteristic	No. (%)	
	In-Network Patients (n = 8418)	Out-of-Network Patients (n = 17 042)
Age at first admission, y		
0-1	3308 (39.3)	6031 (35.4)
2-5	2120 (25.2)	3690 (21.7)
6-11	1570 (18.7)	3412 (20.0)
≥12	1420 (16.9)	3909 (16.9)
Sex		
Female	3869 (46.0)	7779 (45.7)
Male	4549 (54.0)	9263 (54.3)
Race		
Black	5694 (67.6)	7167 (42.1)
White	1329 (15.8)	5226 (30.7)
Asian	219 (2.6)	473 (2.8)
Other	1176 (14.0)	4176 (24.5)
Ethnicity		
Non-Hispanic	7644 (90.8)	14 229 (83.5)
Hispanic	774 (9.2)	2813 (16.5)
Complex chronic conditions, No.^a^		
0	5896 (70.0)	11 126 (65.3)
1	1612 (19.2)	3521 (22.7)
≥2	910 (10.8)	2395 (14.1)

^a^ Defined as medical conditions expected to last longer than 12 months and affecting multiple organ systems or, if affecting 1 organ system, requiring hospitalizations, organ transplantation, or technology dependence.

**Figure 2.**
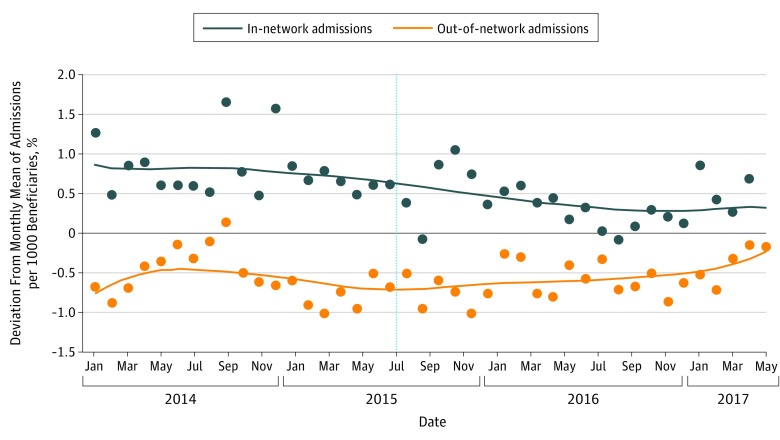
Monthly Inpatient Admission Rate per 1000 Medicaid Beneficiaries, by Child’s Receipt of In-Network Primary Care, Between January 2014 and June 2017 Blue dots represent the monthly inpatient admission rate per 1000 in-network Medicaid beneficiaries. The blue line is a locally weighted Lowess overlay of in-network admissions (bandwidth, 0.6). Orange dots represent the monthly inpatient admission rate per 1000 out-of-network Medicaid beneficiaries. The orange line is a locally weighted Lowess overlay of out-of-network admissions (bandwidth, 0.6). Monthly rates were adjusted using monthly dummy variables to account for naturally occurring variations in admissions and bed-days over the course of the year. The vertical line indicates the start of the intervention.

Figure 2 shows trends in monthly rates of admissions for in-network and out-of-network patients. Accounting for monthly variation, mean admission rates among in-network and out-of-network patients appeared qualitatively similar before program launch in July 2015 (in-network, 4.79 [95% CI, 4.53 to 5.06] monthly admissions per 1000 beneficiaries; out-of-network, 3.44 [95% CI, 3.18 to 3.71] monthly admissions per 1000 beneficiaries) (Table 2). After program launch, mean admission rates for the out-of-network patients remained largely stable, while admission rates for in-network patients decreased (in-network, 4.37 [95% CI, 4.10 to 4.63] monthly admissions per 1000 beneficiaries; difference, −0.43 [95% CI, −0.63 to −0.22] monthly admissions per 1000 beneficiaries; out-of-network, 3.40 [95% CI, 3.16 to 3.67] monthly admissions per 1000 beneficiaries; difference, −0.04 [95% CI, −0.24 to 0.17] monthly admissions per 1000 beneficiaries) (Table 2).

**Table 2.** Difference-in-Differences Estimates of Health Care Utilization Among Medicaid Patients Who Received In-Network Primary Care Compared With Out-of-Network Medicaid Patients^a^

Outcome Measure	Mean (95% CI)			Unadjusted Effect^b^		Adjusted Effect^b^^,^^c^	
	Preintervention Period	Postintervention Period	Difference	Coefficient (95% CI)	*P* Value	Coefficient (95% CI)	*P* Value
Monthly admissions per 1000 beneficiaries, No.							
In-network	4.79 (4.53 to 5.06)	4.37 (4.10 to 4.63)	−0.43 (−0.63 to −0.22)	−0.39 (−0.68 to −0.10)	.009	−0.54 (−0.95 to −0.13)	.01
Out-of-network	3.44 (3.18 to 3.71)	3.40 (3.16 to 3.67)	−0.04 (−0.24 to 0.17)				
Monthly bed-days per 1000 beneficiaries, No.^d^							
In-network	16.12 (14.93 to 17.32)	15.20 (14.03 to 16.37)	−0.92 (−1.85 to 0)	−2.20 (−3.49 to −0.90)	.001	−3.25 (−5.04 to −1.46)	.001
Out-of-network	15.76 (14.57 to 16.96)	17.03 (15.87 to 18.21)	1.27 (0.35 to 2.20)				

^a^ Calculated from 84 group-month observations.

^b^ All difference-in-differences regression models estimated utilization outcomes and included monthly dummy variables to adjust for naturally occurring variations in admissions and bed-days during the year.

^c^ Adjusted models also included sex, race/ethnicity, and number of children with at least 2 complex chronic conditions to account for differential growth in children who were in-network and presented with increasingly complex medical conditions over time.

^d^ Computed based on length of stay winsorized at the 99th percentile to minimize the effect of extreme outliers.

**Figure 3.**
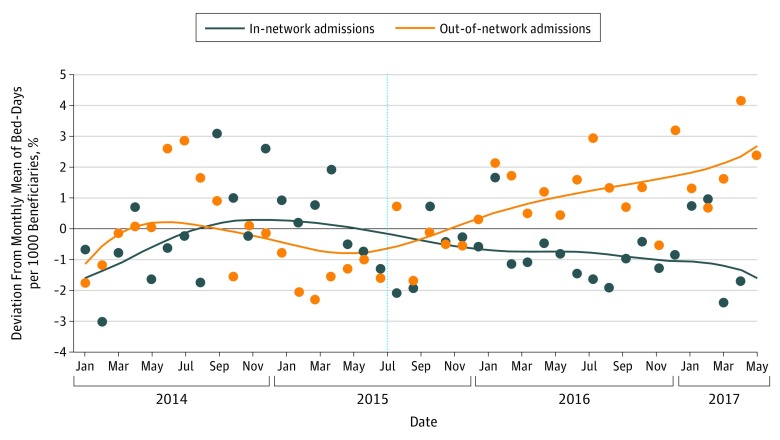
Monthly Inpatient Bed-Days per 1000 Medicaid Beneficiaries, by Child’s Receipt of In-Network Primary Care, Between January 2014 and June 2017 Blue dots represent the monthly inpatient bed-days per 1000 in-network Medicaid beneficiaries. The blue line is a locally weighted Lowess overlay of in-network bed-days (bandwidth, 0.6). Orange dots represent the monthly inpatient bed-days per 1000 out-of-network Medicaid beneficiaries. The orange line is a locally-weighted Lowess overlay of out-of-network bed-days (bandwidth, 0.6). Monthly rates were adjusted using monthly dummy variables to account for naturally occurring variations in admissions and bed-days over the course of the year. Bed-day rates were computed based on length-of-stay winsorized at the 99th percentile to minimize the effect of extreme outliers. The vertical line indicates the start of the intervention.

Figure 3 shows mean monthly number of bed-days for in-network and out-of-network patients, before and after program implementation. Although there was greater variation in bed-days compared with counts of admissions, overall there was a modest decrease in mean monthly bed-days among in-network patients compared with an increase in mean bed-days among out-of-network patients (in-network, 16.12 [95% CI, 14.93 to 17.32] vs 15.20 [14.03 to 16.37] monthly bed-days per 1000 beneficiaries; difference, −0.92 [95% CI, −1.85 to 0]; out-of-network, 15.76 [95% CI, 14.57 to 16.96] vs 17.03 [95% CI, 15.87 to 18.21] monthly bed-days per 1000 beneficiaries; difference, 1.27 [95% CI, 0.35 to 2.20) (Table 2). In formal tests of parallel preintervention trends for the in-network and out-of-network populations, we were unable to reject the null hypothesis of parallel trends for both admissions (*F*_2,63_ = 1.818; *P* = .17) and patient bed-days (*F*_2,63_ = 1.772; *P* = .18).

The difference-in-differences estimates reported in Table 2 confirm the patterns evident in Figure 2 and Figure 3. The reduction of monthly admissions among in-network patients was greater by 0.39 (95% CI, 0.10-0.68) admissions per 1000 beneficiaries compared with out-of-network patients (*P* = .009). In-network patients similarly experienced a decrease of 2.20 (95% CI, 0.90-3.49) bed-days per 1000 beneficiaries compared with out-of-network patients (*P* = .001).

Controlling for the monthly proportion of children with at least 2 CCCs who were in the in-network group, the reduction in monthly admissions among in-network patients increased to 0.54 (95% CI, 0.13-0.95) per 1000 beneficiaries compared with out-of-network patients (*P* = .01). Similarly, the reduction in monthly bed-days among in-network patients increased to 3.25 (95% CI, 1.46-5.04) bed-days per 1000 beneficiaries compared with out-of-network patients (*P* = .001) (Table 2). Annualized, these differences could translate to a reduction of 3600 bed-days for a population of 93 000 children eligible for Medicaid.

## Discussion

This quality improvement study demonstrated that a targeted population health management strategy providing broad registry-based tools across a health system’s Medicaid-enrolled primary care population, alongside more precise integrated care team interventions for children with higher risk within the cohort, was associated with a large aggregate decrease in admissions and bed-days to the parent hospital. Our findings add to existing literature on population health programs because of our strong quasi-experimental design and the novel application of a unified population health improvement framework that stratified the intensity of resources across several risk groups simultaneously.

As pediatric health systems seek to integrate population health programs, few robust evaluations of such initiatives exist, particularly among children enrolled in Medicaid.^16,24^ Prior studies^25,26,27^ revealed a potential benefit of targeted care management programs for medically complex children and multicomponent improvement collaborative for children with asthma, while others had more equivocal or modest results. A global capitation model^24^ for children enrolled in Medicaid demonstrated similarly mixed results, with restrained growth in Medicaid costs compared with private insurance and single-arm improvement for some quality metrics but no reduction in hospitalization rates for asthma.

The targeted approach described in our study provides a scalable and cost-effective strategy for other health systems as they determine the size of their investments and prioritization of programs for population health initiatives.^28^ Although other health systems may focus interventions on patients with different clinical profiles, the success of this population-level intervention was driven by financial investments in the development of integrated care teams, alignment between clinical and quality improvement methods, and the wide availability of EHR tools. Our approach used interdisciplinary teams and quality improvement methods to align clinical workflow across team members using tools within the EHR. Technology developed in recent years to support interdisciplinary clinical teams offers a lens through which the Medicaid program and pediatric health systems can assess costs and benefits of programmatic interventions targeted at patients with high risk. The magnitude of the bed-day reduction, when scaled to the monthly attribution of all in-network patients who were enrolled in Medicaid, was estimated to exceed 10 beds daily or 3600 bed-days annually, representing a substantial potential savings of inpatient costs to the Medicaid program.

Although our results are promising, important conditions promoting success may not be present in all health systems. The health system in this study had advantages in managing its patients with the highest risk through a primary care model, given its employed physician staffing model and unified EHR platform across its network practices, emergency department, and hospital. Nevertheless, despite barriers in practice integration or EHRs that other health systems may face, the quality improvement methods used to develop these targeted interventions can help standardize population health management approaches.

We examined the value of this intervention from the perspective of the health system. The magnitude of savings achieved for reducing inpatient bed-days was nearly 6-fold the investment that the health system made in hiring integrated care teams and expanding access to registry and reporting tools. Ambulatory utilization also increased. However, from the perspective of the payer, the degree to which increased ambulatory utilization and potential home nursing or pharmacy services might have attenuated economic savings remains uncertain.^29^

### Limitations

This study has limitations. First, while we included time-dependent adjustment for medical complexity, there may have been residual unobservable case-mix differences between the in-network and out-of-network populations. To some degree, the difference-in-differences analysis is robust to selection bias in comparison groups, so long as selection differences do not vary over time. For example, our team considered that the control group might have increasingly ventured outside the health system over time, but separate demographic characteristic analyses revealed consistent trends in neighborhoods nearest and furthest from the hospital (data not shown). Second, we did not have information on children’s admissions to hospitals outside our network. However, preferential use of a health system’s inpatient facilities by children within its primary care practices might be expected. If selection difference increased over time, we might have underestimated the consequences of the population management programs. Third, while we have a precise estimate of enrollment in the primary care network, this may not have fully captured the magnitude of children who were out-of-network to the health system. To account for this, we used state Medicaid enrollment files to estimate changes during the study period in the region’s census of the Medicaid-enrolled pediatric population who were not attributed to the health system’s practices. Our team believed these trends reflected increasing number of out-of-network children in the greater metropolitan area, even if the monthly estimates themselves may not have been precise.

## Conclusions

Market consolidation, increasing regional responsibility for hospital and subspecialty care, and growing Medicaid enrollment among patients have created new challenges for large pediatric health systems that provide care to children with medical complexity. This study provided quasi-experimental data for how population management, imbued with interdisciplinary teams, quality improvement, and information technology, can help large pediatric health systems respond to the needs of an increasing, medically complex, Medicaid-enrolled population and mitigate capacity challenges by reducing inpatient hospitalization among the managed population.

## Notes

Supplement. **eFigure.** Monthly Number of Patients With 2 or More Complex Chronic Conditions, by Child’s Receipt of In-Network Primary Care, January 2014 to July 2017
