# Supplementary material for: Association of a Targeted Population Health Management Intervention with Hospital Admissions and Bed-Days for Medicaid-Enrolled Children
Source: JAMA Netw Open. 2019 Dec 27;2(12):e1918306. doi: 10.1001/jamanetworkopen.2019.18306 (PMC6991308; doi:10.1001/jamanetworkopen.2019.18306)
Supplement: Supplement. — eFigure. Monthly Number of Patients With 2 or More Complex Chronic Conditions, by Child’s Receipt of In-Network Primary Care, January 2014 to July 2017 [file jamanetwopen-2-e1918306-s001.pdf]

Supplementary Online Content

Rubin DM, Kenyon CC, Strane D, et al. Association of a targeted population health management intervention with hospital admissions and bed-days for Medicaid-enrolled children. *JAMA Netw Open*. 2019;2(12):e1918306. doi:10.1001/jamanetworkopen.2019.18306

eFigure. Monthly Number of Patients with 2 or More Complex Chronic Conditions, by Child's Receipt of In-Network Primary Care, January 2014 to July 2017

This supplementary material has been provided by the authors to give readers additional information about their work.

© 2019 Rubin DM et al. *JAMA Network Open.*

eFigure. Monthly Number of Patients with 2 or More Complex Chronic Conditions[a], by Child's Receipt of In-Network Primary Care, January 2014 to July 2017

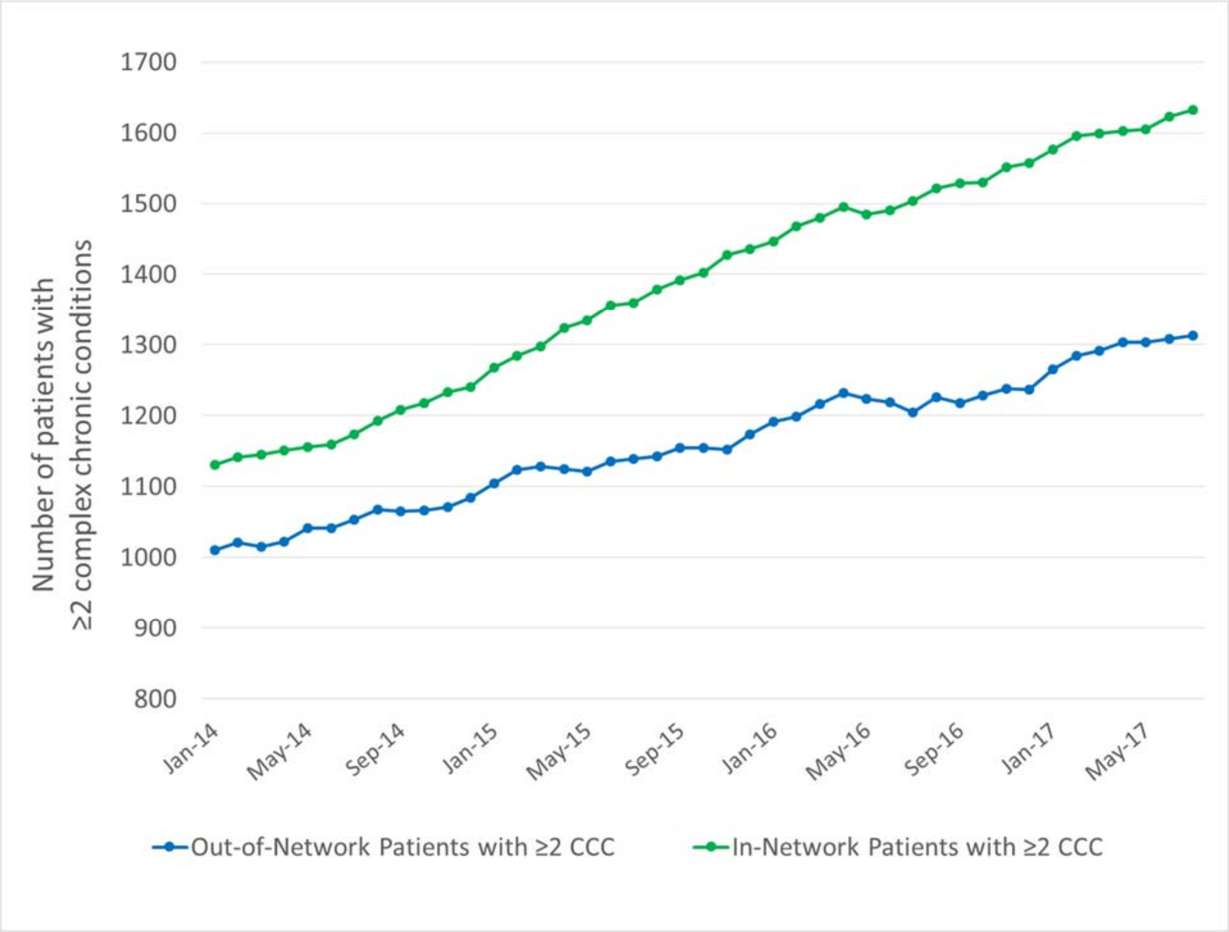

[a] Complex chronic conditions are defined as medical conditions expected to last longer than 12 months, affecting multiple organ systems or one organ system but requiring hospitalizations, or necessitating organ transplantation or technology dependence.

© 2019 Rubin DM et al. *JAMA Network Open.*
